# Label-free intraoperative nerve detection and visualization using ratiometric diffuse reflectance spectroscopy

**DOI:** 10.1038/s41598-023-34054-6

**Published:** 2023-05-10

**Authors:** Graham A. Throckmorton, Ezekiel Haugen, Giju Thomas, Parker Willmon, Justin S. Baba, Carmen C. Solórzano, Anita Mahadevan-Jansen

**Affiliations:** 1 Department of Biomedical Engineering, Vanderbilt University, Nashville, TN 37235 USA; 2 Vanderbilt Biophotonics Center, Vanderbilt University, Nashville, TN 37235 USA; 3 Yaya Scientific LLC, Nashville, TN 37203 USA; 4 Department of Surgery, Vanderbilt University Medical Center, Nashville, TN 37232 USA; 5 Department of Otolaryngology, Vanderbilt University Medical Center, Nashville, TN 37232 USA; 6 Department of Neurological Surgery, Vanderbilt University Medical Center, Nashville, TN 37232 USA

**Keywords:** Biophotonics, Translational research

## Abstract

Iatrogenic nerve injuries contribute significantly to postoperative morbidity across various surgical disciplines and occur in approximately 500,000 cases annually in the US alone. Currently, there are no clinically adopted means to intraoperatively visualize nerves beyond the surgeon’s visual assessment. Here, we report a label-free method for nerve detection using diffuse reflectance spectroscopy (DRS). Starting with an in vivo rat model, fiber- and imaging-based DRS independently identified similar wavelengths that provided optimal contrast for nerve identification with an accuracy of 92%. Optical property measurements of rat and human cadaver tissues verify that the source of contrast between nerve and surrounding tissues is largely due to higher scattering in nerve and differences in oxygenated hemoglobin content. Clinical feasibility was demonstrated in patients undergoing thyroidectomies using both probe-based and imaging-based approaches where the nerve were identified with 91% accuracy. Based on our preliminary results, DRS has the potential to both provide surgeons with a label-free, intraoperative means of nerve visualization and reduce the incidence of iatrogenic nerve injuries along with its detrimental complications.

## Introduction

Inadvertent nerve damage, also termed iatrogenic nerve injury (INI), yields unfavorable post-operative outcomes for patients and can pose a major challenge for surgeons. In complex surgeries that require extensive and precise dissection, such as mastectomies and prostatectomies, INIs occur in 40–60% of cases^1–4^. Moreover, interpatient anatomic variability can further hamper a surgeon’s ability to locate and identify nerves whether due to reoperations, pathologies, or natural anatomic deviations^5–7^. Consequently, between 400,000 and 600,000 INIs occur each year in the US^8–10^. Depending on which nerve is damaged and to what extent, the effects of INI range from loss of sensation and tingling to paralysis and morbidity^11–16^. As a result, INIs often lead to expensive malpractice lawsuits against healthcare institutions^17^. For these reasons, different means of preventing and managing nerve damage have been developed and adopted.

Currently, intraoperative nerve monitoring is the main approach for functional nerve assessment where the risk of nerve injury is high, or the effects of the nerve damage are severe. Nerve monitoring, however, does not protect nerves but rather only alerts clinicians once damage has occurred. Hence, recent efforts have focused on visualizing nerves within the surgical field to help prevent nerve damage altogether. Clinically, nerve imaging is primarily performed with ultrasound or magnetic resonance imaging^5,18–21^. While some procedures use ultrasound intraoperatively^22^, these methods only visualize nerves preoperatively. Consequently, there are no real-time means to identify or visualize nerves intraoperatively. Moreover, ultrasound and magnetic resonance imaging struggle to generate sufficient contrast to distinguish nerve from surrounding tissues and structures^19,23^. Thus, there is a clinical need to develop an intraoperative method to visualize nerves.

In order to provide adequate spatial resolution for nerve visualization, a variety of optical techniques are being evaluated including photoacoustic imaging^24,25^, optical coherence tomography^26,27^, fluorescence imaging^28–32^, non-linear imaging^33–35^, and polarization imaging^36–39^. Many of these techniques are limited by expensive and/or technically complex instrumentation, intricate image and/or postprocessing, slow imaging speeds, or the administration of exogenous contrast agents. The use of contrast agents introduces inherent difficulties such as associated allergic reactions, the timing of administration, or highly variable pharmacokinetics. To overcome these obstacles, we propose using diffuse reflectance spectroscopy (DRS) for intraoperative nerve detection and visualization. DRS maintains the spatial resolution of optical imaging while requiring simple and relatively inexpensive instrumentation and minimal postprocessing. DRS is a label-free optical technique in which a sample is exposed to a broad range of wavelengths, usually within the visible to near infrared region, and the backscattered light is collected. This signal is dependent on the intrinsic optical properties of the sample including tissue absorption and scattering^40^. Since these optical properties largely depend on the presence and concentration of biological chromophores and scatterers within various tissues, DRS is well suited to differentiate tissues based on their biomolecular composition. Moreover, the signal provided by DRS is much stronger than that produced by endogenous fluorescence enabling the use of low-cost detectors and simplified system design. Previous groups that have utilized DRS to discriminate nerve from surrounding tissues have relied on (i) complex, whole spectra analyses based on simplified assumptions, (ii) expensive infrared spectrometers to extend the spectral range further into the shortwave infrared region, or (iii) only investigated the use of fiberoptic DRS^41–48^.

In this study, we aim to develop a label-free means to detect and visualize nerves intraoperatively using DRS. Since surgeons differ on their preferences for fiberoptic- or imaging-based approaches and each has their advantages, the efficacy of both implementations are assessed for intraoperative nerve detection and visualization. Optimal wavelengths for ratiometric nerve discrimination were identified using both approaches in an in vivo rat model. Optical property measurements were subsequently made on rat and human cadaver tissue to verify the optical sources of contrast. The ratiometric approach was then tested in patients undergoing thyroidectomies to validate the clinical effectiveness of both probe-based and imaging-based DRS for intraoperative nerve localization.

## Results

### Identification of optimal wavelengths for nerve discrimination

**Figure 1:**
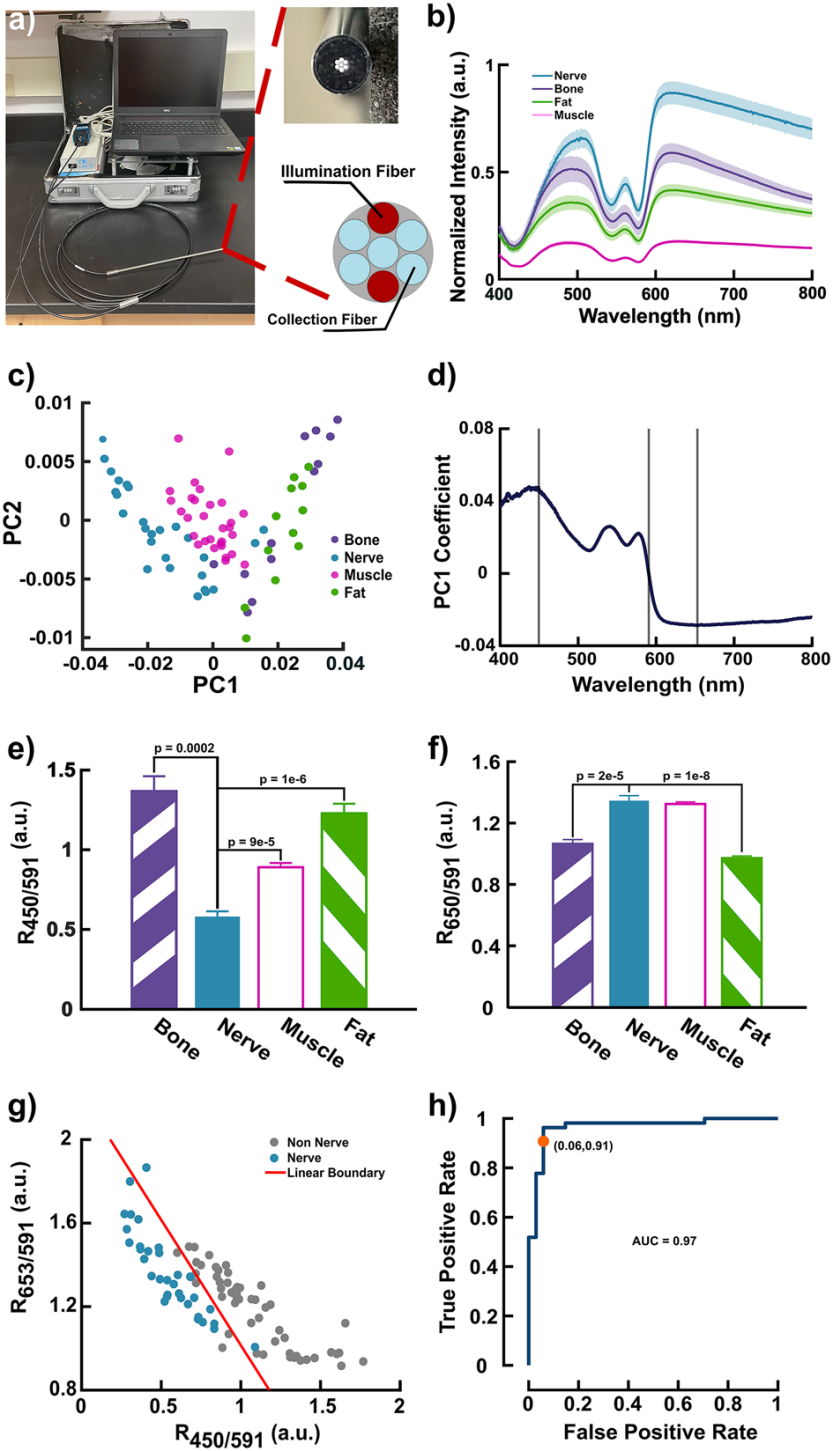
Discriminatory wavelength selection using probed-based DRS of in vivo rat tissues. (**a**) Image of portable probe-based DRS system along with the fiber configuration of the probe. (**b**) Average DR spectra from bone, fat, muscle, and nerve. (**c**) Plot of principle components 1 and 2 using spectra collected from bone, fat, muscle, and nerve. (**d**) Loading coefficients of principle component 1 versus wavelength. The three bands indicate wavelengths corresponding to the maximum and minimum loading coefficients at 450 and 653 nm along with the zero coefficient at 591 nm. (**e**) Ratio of 450–591 nm (${R}_{450/591}$) of the four considered tissue types. (**f**) Ratio of 653–591 nm (${R}_{653/591}$) of the four considered tissue types. Statistical tests for determining significance and reported $p$-values were performed and calculated using a one-way analysis variance (ANOVA) followed by a Tukey’s test. All error bars represent the mean ± standard error of the mean (SEM). $n=13$ rats. (**g**) Scatter plot of ${R}_{653/591}$ versus ${R}_{450/591}$ plotted with the resultant linear separator from linear discriminant analysis separating nerve from all other tissues. (**h**) Receiver operator characteristic (ROC) curve of linear discriminant analysis using ${R}_{653/591}$ and ${R}_{450/591}$.

An in vivo rat sciatic nerve preparation was used to determine optimal wavelengths for nerve detection using a portable fiber-based DRS system (Fig. 1a). The fiber-based system consists of a halogen-tungsten lamp as the excitation source, a visible to near infrared spectrometer, a laptop to control components and display spectra, and a fiberoptic probe arranged in a six-around-one configuration for light delivery and collection. Of the seven fibers, two are illumination fibers while the remainder serve as collection fibers to ensure adequate signal collection (Fig. 1a). During each animal experiment, spectra were collected from bone, nerve, muscle, and fat tissue in vivo. Background spectra were taken with the lamp turned off and the probe pressed gently against the tissue. Reference spectra were collected at the end of each experiment using a 99% diffuse reflectance standard and used to account for the spectral distribution of the lamp.

Average spectra of the four considered tissues are plotted in Fig. 1b. Among these tissues, nerve has the highest overall diffuse reflectance followed by that of bone. Principle component analysis (PCA) was performed to identify which wavelengths could best discriminate nerve from the surrounding tissues. Results show that PC1 and PC2 account for 94% of the variance while PC1 alone is responsible for 82% of the variance. Plotting PC1 against PC2 allows nerve tissue to be differentiated from the other tissues (Fig. 1c). Hence, the PC1 loadings were used to identify optimal wavelengths for nerve detection (Fig. 1d). Interestingly, PC1 loading coefficients resemble a near mirror image of the tissue spectra. The maximum positive and minimum negative loading coefficients occur at 450 and 653 nm respectively and were selected as candidates for discrimination. The wavelength that contributed minimally to the overall variance (i.e. the loading coefficient closest to 0), 591 nm, was chosen as a normalizing factor. Ratioing the spectral intensity at 450–591 nm (${R}_{450/591}$) and 653–591 nm (${R}_{653/591}$) yielded statistically distinct distributions. Using ${R}_{450/591}$, the separation between nerve and the other tissues is statistically significant ($p<2*{10}^{-4}$; Fig. 1e). Using ${R}_{653/591}$, however, nerve was only statistically distinct from fat and bone ($p<2*{10}^{-5}$; Fig. 1f). A binary classification of nerve tissue (i.e. observations were classified as nerve or non-nerve) was then performed via linear discriminant analysis (LDA) using the two ratios (Fig. 1g). The prediction from LDA achieved a sensitivity and specificity of 91% and 94% respectively after k-fold cross validation ($k=5$) for an overall accuracy of 92% with the resultant ROC curve having an area under the curve (AUC) of 0.97 as shown in Fig. 1h.

### Validation of imaging-based DRS for nerve visualization via hyperspectral imaging

**Figure 2:**
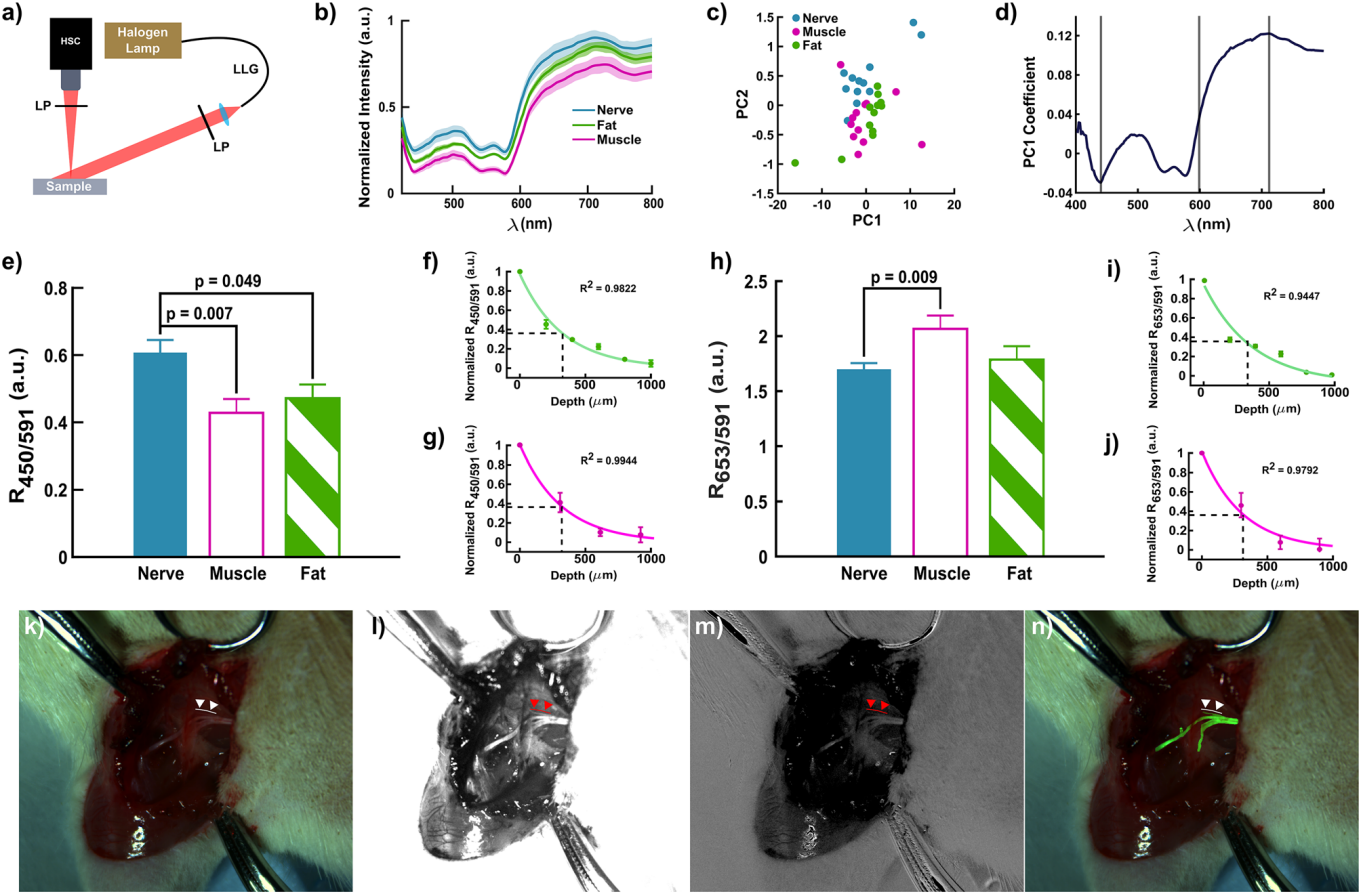
Hyperspectral imaging identifies similar discriminatory wavelengths enhancing nerve visualization. (**a**) Schematic of hyperspectral imaging system. LLG-liquid light guide, LP-Linear Polarizer, HSC-Hyperspectral Camera. (**b**) Average spectra from regions corresponding to nerve, muscle, and fat. Shaded regions represent the standard deviation. (**c**) Plot of PC2 vs. PC1 using the nerve, muscle, and fat spectra collected using the hyperspectral camera. (**d**) Loading coefficients of PC1 plotted against wavelength. The three gray bands indicate wavelengths corresponding to the maximum and minimum coefficients at 452 nm and 712 nm along with the zero coefficient at 599 nm. (**e**) ${R}_{450/591}$ of nerve, muscle, and fat. Bars represent the mean ± SEM. (**f**) ${R}_{450/591}$ nerve signal as it decays under varying thicknesses of fat normalized to maximum signal of the uncovered nerve. Dashed lines mark thickness of fat at which ${R}_{450/591}$=$1/e$. Error bars are the standard deviation. (**g**) ${R}_{450/591}$ nerve signal as it decays under varying thicknesses of muscle normalized to maximum signal of the uncovered nerve. Dashed lines mark thickness of fat at which ${R}_{450/591}$=$1/e$. Error bars are the standard deviation. (**h**) ${R}_{653/591}$ of nerve, muscle, and fat. Bars represent the mean ± SEM. (**i**) ${R}_{653/591}$ nerve signal as it decays under varying thicknesses of fat normalized to maximum signal of the uncovered nerve. Dashed lines mark thickness of fat at which ${R}_{653/591}$=$1/e$. Error bars are the standard deviation. (**j**) ${R}_{653/591}$ nerve signal as it decays under varying thicknesses of muscle normalized to maximum signal of the uncovered nerve. Dashed lines mark thickness of fat at which ${R}_{653/591}$=$1/e$. Error bars are the standard deviation. (**k**) White light image of rat sciatic nerve preparation in vivo. Triangles indicate the location of a 370 µm diameter posterior cutaneous nerve. (**l**) ${R}_{450/591}$ image of the same animal. (**m**) ${R}_{653/591}$ image of the same animal. (**n**) Post-processed image (green) superimposed on the white light image of nerve generated using the ${R}_{450/591}$ and ${R}_{653/591}$. Statistical tests for determining significance and reported $p$ -values were performed and calculated using a one-way analysis variance (ANOVA) followed by a Tukey’s test. $n=12$ rats.

Diffuse reflectance imaging was also performed to enable nerve visualization. To verify if the wavelength selection determined with the probe-based system would hold for imaging systems, imaging was conducted with the system depicted in Fig. 2a. White light from a halogen lamp was coupled into a liquid light guide to obliquely illuminate the field of view to reduce specular reflections. Specular reflectance was further minimized with linear polarizers placed at the output of the liquid light guide and prior to the collection optics of the camera in a crossed-polarized orientation. The measured irradiance at the sample surface was $0.2\;{\text{mW}}/{\text{cm}}^{2}$. As with the fiber-based system, rat sciatic nerves as well as surrounding tissue were imaged in vivo from 400 to 800 nm.

As seen in Fig. 2b, nerve again exhibited the highest overall diffuse reflectance followed by fat and then muscle. Using the same statistical analyses, PCA was performed on the spectra obtained with the imaging system. Figure 2c shows the separation obtained between nerve and all other tissues when PC2 is plotted against PC1. Of the explained variance, 98% was described by PC1 and PC2. Individually, PC1 accounted for 95% of the variance and thus was used again to identify the best possible wavelengths for nerve detection. Unlike the probe-based loading coefficients, the curve of the loading coefficients of PC1 derived from imaging data resembled the spectra themselves (Fig. 2d). Nonetheless, wavelengths identified by the imaging system were similar to those identified by the fiber-based system: 452 nm as the minimum coefficient, 712 nm as the maximum coefficient, and 599 nm as the zero coefficient.

Since the spectral resolution of the imaging system was ~ 10 nm, 450 and 591 nm wavelengths were used for ratiometric analysis (since they fell within the spectral resolution of the system) to facilitate comparison between systems. Though PCA identified 712 nm rather than 653 nm for the maximum coefficient, there was no loss in statistical significance using ${R}_{653/591}$ over ${R}_{712/591}$ (Fig. S1). The difference between the ${R}_{450/591}$ of nerve, muscle and fat remained statisitically significant ($p=0.007$ and $p=0.049$ respectively; Fig. 2e). While the difference between the ${R}_{653/591}$ of nerve and muscle is significant ($p=0.009$), the difference between nerve and fat was not (Fig. 2h).

The depth of detection for each ratio was also determined by overlaying ex vivo nerves with layers of fat and muscle of various thicknesses. To quantify the decay in the ratiometric signal as a function of depth, the mean intensity from pixels cooresponding to nerve were divided by the mean inensity of pixels from either muscle or fat. For ${R}_{450/591}$, the depth at which the ratio decrease by $1/e$ was 327 µm for nerve overlaid with fat and 313 µm for nerve overlaid with muscle respectively (Fig. 2f, g). For ${R}_{653/591}$, the $1/e$ depth of detection was 383 µm and 311 µm for fat and muscle respectively (Fig. 2f, g).

Spectral images of the surgical field at the 3 wavelengths were extracted and the two ratiometric images (${R}_{450/591}$ and ${R}_{653/591}$) were calculated and superimposed to provide enhanced visualization of the nerves in the surgical field. Compared to the white light image in Fig. 2k, the ${R}_{450/591}$ and ${R}_{653/591}$ images (Fig. 2l and m respectively) successfully highlight the branches of the sciatic nerve including the 370 µm diameter posterior cutaneous nerve (indicated by the triangles in the figure). Both ratiometric images were then thresholded, summed, and binarized to create the green outline of nerve tissue overlaid onto the white light image as shown in Fig. 2n.

### Determining sources of contrast at identified wavelengths

**Figure 3:**
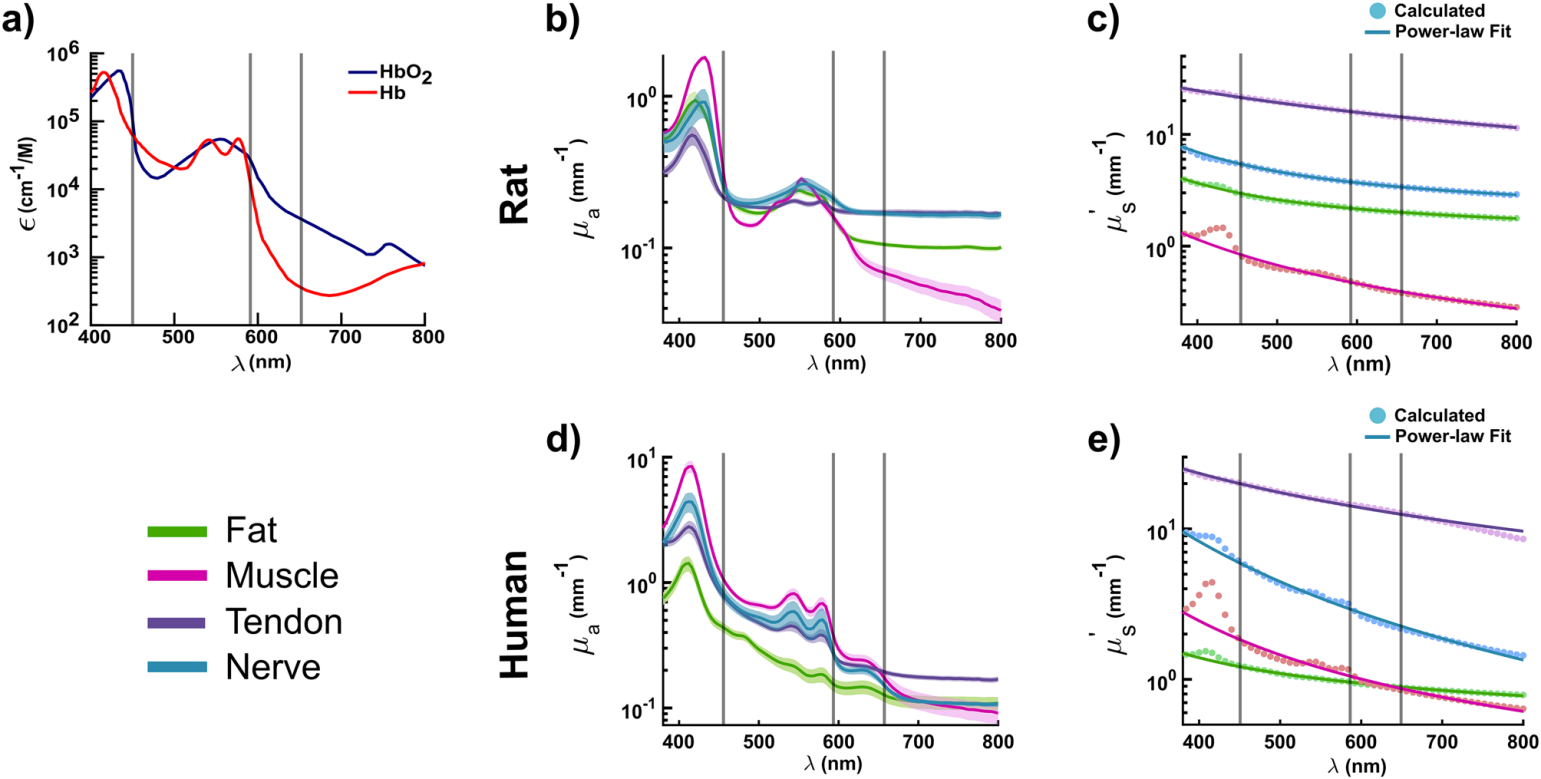
Optical properties of rat and human tissues. (**a**) Molar extinction coefficient of oxygenated (HbO_2_) and deoxygenated (Hb) hemoglobin as a function of wavelength using data from^49^. Gray lines indicate the three key identified wavelengths. (**b**) Absorption coefficient spectra of rat nerve, muscle, fat, and tendon. Shaded error bars are the standard error. (**c**) Reduced scattering coefficient spectra of rat nerve, muscle, fat, and tendon. Data points are the calculated reduced scattering coefficient values which were fit to the power-law Mie-Rayleigh scatting equation (solid lines). (**d**) Absorption coefficient spectra of human nerve, muscle, fat, and tendon. Shaded error bars are the standard error. (**e**) Reduced scattering coefficient spectra of human nerve, muscle, fat, and tendon. Data points are the calculated reduced scattering coefficient values which were fit to the power-law Mie-Rayleigh scattering equation (solid lines). ($n=5$).

In order to determine the source of contrast that allows nerves to be distinguished from the other tissues at the key wavelengths in both the probe and imaging DRS systems, optical properties of the tissues and biological chromophores were evaluated. First, oxygenated and deoxygenated hemoglobin extinction coefficient spectra were examined as the dominant chromophores in the visible region (Fig. 3a)^49^. Interestingly, both 450 and 591 nm correspond to isosbestic points between the hemoglobin spectra. While this supports the use of 591 nm as a normalizing factor, the isosbestic point at 450 nm leads to the hypothesis that the source of contrast at this wavelength is primarily due to scattering. On the other hand, 653 nm occurs in a region where the two hemoglobin spectra differ considerably. Hence, the source of contrast at this wavelength is hypothesized to be due to a difference in tissue oxygenation and possibly metabolic state. To confirm these conclusions, optical properties were measured on each of the studied tissues.

Total reflectance and transmittance were measured from 400 to 800 nm on fresh ex vivo rat and thawed human cadaver nerve, muscle, fat, and tendon using a dual-beam single integrating sphere spectrophotometer. The absorption coefficient (${\mu }_{a}$) and reduced scattering coefficient ($\mu_{s}^{\prime }$) of each tissue were then calculated via the inverse adding doubling method^50^. The accuracy of the procedures used to obtain the optical properties was validated using 1.1 µm polystyrene spheres with known scattering properties and methylene blue, a well characterized chromophore (Fig. S2)^51^. The absorption spectra of the four rat tissues share the same isosbestic point near 450 nm as seen in hemoglobin extinction coefficient spectra (Fig. 3b). Additionally, rat muscle, fat, and nerve absorption spectra also converge near 591 nm with the exception of muscle. At 653 nm, the nerve absorption spectrum diverges from that of muscle and fat.

Similar comparisons were also made using the optical properties from human cadaver tissues to ascertain the similarities and differences between human and rat tissues. The variability in the absorption spectra of human cadaver tissues was greater than the equivalent rat tissues save that of muscle (Fig. 3d). The isosbestic point at 450 nm was only observed in rat tissues and is not present in the human tissue absorption spectra. The isosbestic point at 591 nm is also is less pronounced in the human tissues. At 591 nm, however, tendon and nerve show overlapping ${\mu }_{a}$ (0.28 and 0.3 mm^-1^ respectively) with muscle tracking closely (0.4 mm^-1^). Human fat and tendon absorption spectra, however, diverge from nerve and fat at 653 nm.

The reduced scattering coefficient of human nerve is considerably higher than that of muscle and fat but lower than tendon across the entire spectral range (Fig. 3c and e). At 450 nm, the difference in $\mu_{s}^{^{\prime}}$ between nerve and the other tissues was greater than at longer wavelengths.

### Verification of wavelength selection in human subjects using probe-based diffuse reflectance spectroscopy

**Figure 4:**
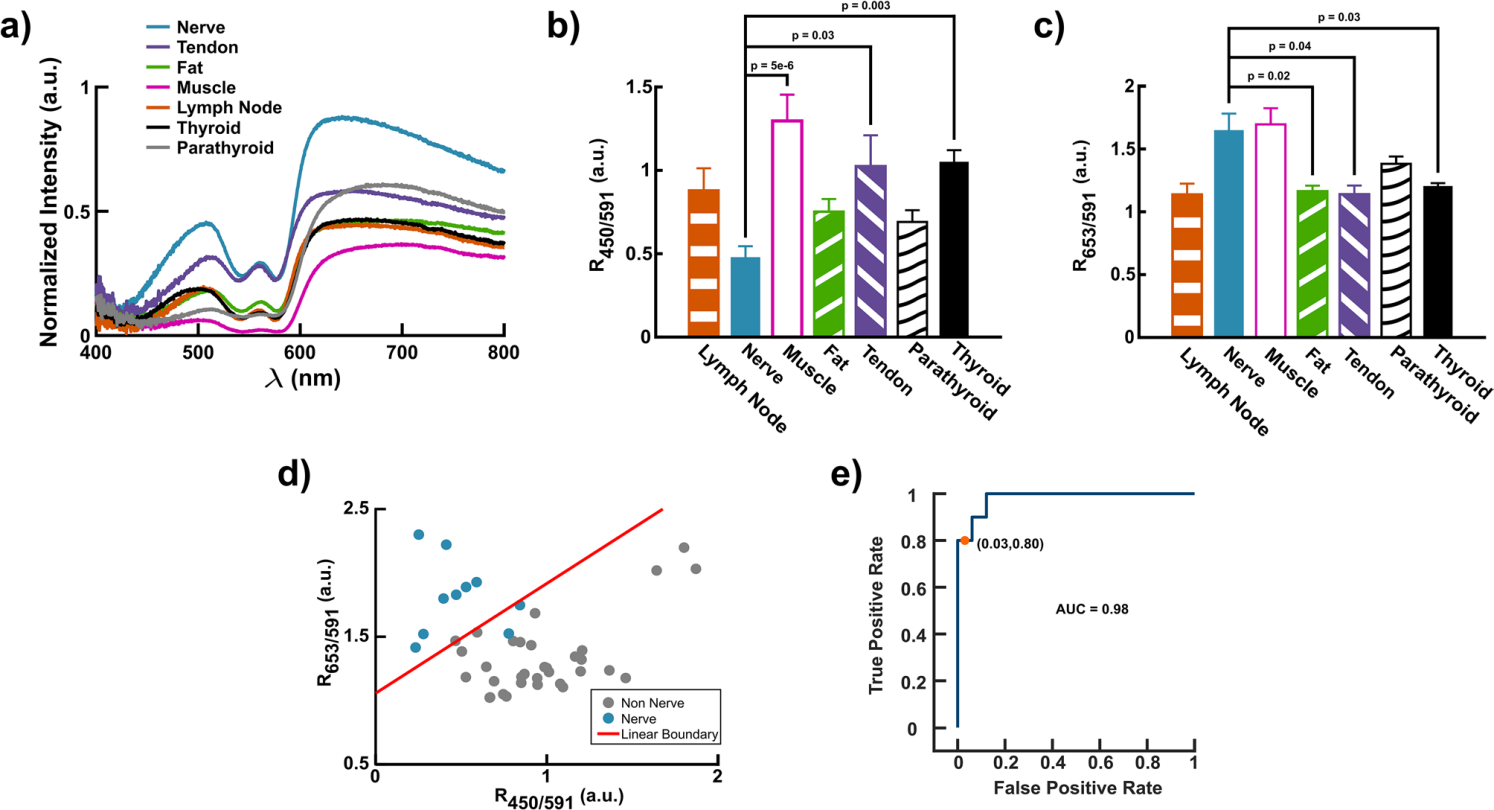
Intraoperative probe-based DRS for nerve detection in human subjects. (**a**) Average diffuse reflectance spectra of human tissues in the neck. (**b**) ${R}_{450/591}$ of nerve, tendon, fat, muscle, lymph node, thyroid, and parathyroid tissues. (**c**) ${R}_{653/591}$ of the seven considered tissue types. (**d**) Scatter plot of ${R}_{653/591}$ versus ${R}_{450/591}$ plotted with the resultant linear separator from LDA. (**h**) Receiver operator characteristic (ROC) curve from LDA using ${R}_{653/591}$ and ${R}_{450/591}$. Statistical tests for determining significance and reported $p$-values were performed and calculated using a one-way analysis variance (ANOVA) followed by a Tukey’s test. All error bars represent the mean ± SEM. $n=6$ patients.

To evaluate the feasibility of DRS to detect nerves from all other tissues in humans in vivo, six patients undergoing thyroidectomy were recruited under IRB approval and with informed consent. The same portable fiber-based DRS system used for the rat studies (Fig. 1a) was utilized, and diffuse reflectance spectra were collected intraoperatively and in vivo from tissues in the neck of the six patients. The list of interrogated tissues was expanded to include nerve, fat, muscle, lymph nodes, tendons, thyroid, and parathyroid glands. Due to the smaller number of patients recruited for the human feasibility study, PCA was not performed; rather spectral ratios at the key wavelengths were compared directly. Additionally, because only two spectra were collected from lymph nodes, these tissue spectra were not included in the statistical analysis for Fig. 4b and c.

Figure 4a shows the average diffuse reflectance spectra of the different tissues from all patients studied. As seen previously, nerves had the greatest reflectance especially at wavelengths longer than 600 nm (Fig. 4a). Applying the same ratiometric approach established in the rat studies, the ${R}_{450/591}$ of nerve was significantly different from that of muscle, tendon, and thyroid tissue (Fig. 4b). Additionally, the ${R}_{653/591}$ of nerve was statistically distinct from fat, tendon, and thyroid tissue (Fig. 4c). Because additional tissues were included in this study, a new LDA model was trained (Fig. 4d). Applying the LDA model previously developed using rat spectra yielded inadequate classification (accuracy = 62%). The linear model trained on human data, however, resulted in an accuracy of 93% with a specificity of 97% and sensitivity of 80% (Fig. 4e).

### Validation of ratiometric spectral imaging for enhanced intraoperative nerve visualization

**Figure 5:**
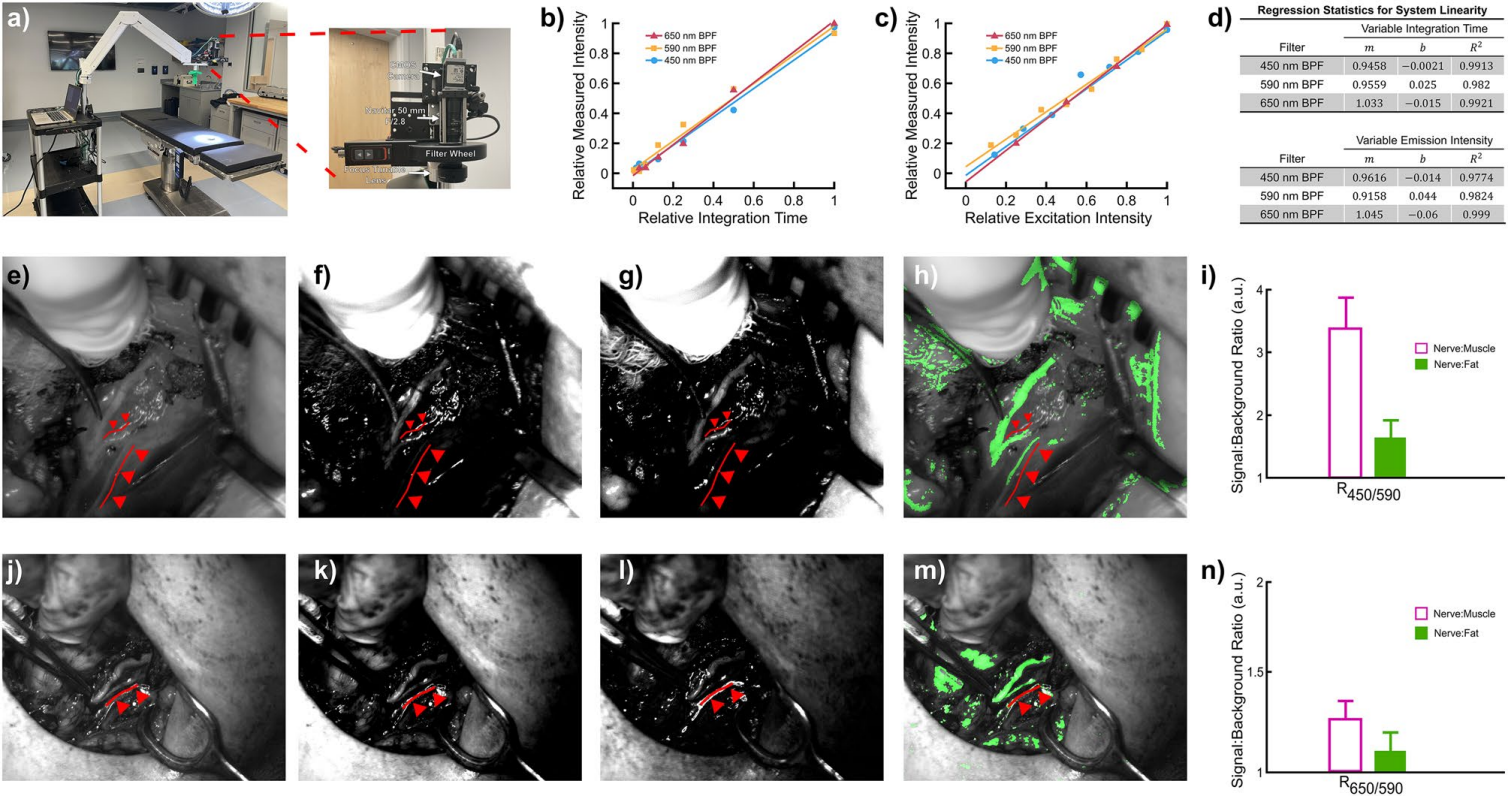
Clinical multispectral imaging for intraoperative nerve visualization during thyroid surgery. (**a**) Picture of clinical MIS with magnified view of the imaging head. (**b**) Linearity measurements of the clinical system for constant excitation intensity and variable integration time for each of the three filters. Scatter points are median values. (**c**) Linearity measurements of the clinical system for constant integration time and variable emission intensity for each of the three filters. Scatter points represent median values. (**d**) Regression statistics for linearity measurements for each of the three filters. (**e**, **j**) White light image of the surgical field in two separate patients. The triangles and surgical forceps indicate the recurrent laryngeal nerve (RLN), the motor branch of the RLN, and/or the esophageal nerve. (**f**, **k**) ${R}_{450/591}$ image of two patients respectively. (**g**, **l**) ${R}_{653/591}$ image of two patients respectively. (**h**, **m**) Post-processed image (green) superimposed on the white light image of surgical field generated using the ${R}_{450/591}$ and ${R}_{653/591}$ images of the two patients. (**i**) Average signal to background ratio (SBR) of nerve to muscle (Nerve:Muscle) and nerve to fat (Nerve:Fat) for ${R}_{450/591}$ across all seven patients (**n**) Average SBR of nerve to muscle (Nerve:Muscle) and nerve to fat (Nerve:Fat) for ${R}_{653/591}$ across all seven patients. Error bars indicate SEM. $n=7$ patients.

To evaluate the performance of the imaging-based approach in patients, a human feasibility was conducted in seven patients undergoing thyroidectomy under IRB approval and with informed consent using a clinical multispectral imaging system (MIS) (Fig. 5a). The long acquisition times required by the hyperspectral camera that was previously used in rat study prohibited its use in the operating room (OR). Therefore, a new system, the MIS, was built which consisted of an articulated arm mounted to a portable cart with the imaging head attached to the free end. The imaging head is comprised of a monochrome camera which collected images of the surgical field at the three previously selected wavelengths with filters centered at 448 ± 10 (450), 590 ± 6 (591), or 655 ± 8 (653) nm housed in a motorized filter wheel. The field of view was approximately 3 cm × 3 cm with the system positioned ~ 50 cm above the operating table so as to remain outside of the sterile field.

Minimizing disruptions to surgical workflow is critical to performing measurements in the OR. This includes eliminating the need to turn off the OR lights during image acquisition. Thus, the clinical MIS was designed to use the existing OR lights as the illumination source. Characterization testing was performed to confirm system linearity using the OR lights as the illumination source and to compensate for disparities in spectral sensitivity. Confirmation of system linearity accounts for two primary benefits. First, wavelength ratios performed on a pixel-by-pixel basis correlate directly with the intensity of the reflectance and consequently are independent of changes in excitation intensity or the topography of the surgical field. Second, system linearity also enables the use of variable integration times for each considered wavelength to ensure that adequate signal is collected for each. Differences in integration times can then be corrected for during image analysis using simple normalization.

To investigate system linearity, a 99% diffuse reflectance standard was imaged in a mock OR equipped with the same OR lights present in the OR where the thyroidectomies were performed. First, the integration time of the camera was varied while the excitation intensity of the OR lights was held constant. Data at every integration time was also collected at different illumination angles. After correcting for the non-zero offset using background subtraction, the measured intensity of the MIS is observed to be linear at each of the three filters/wavelengths (Fig. 5b, ${R}^{2}>0.98$). As the fitting was applied to all the intensity values acquired regardless of the orientation of the OR lights, the MIS exhibited minimal dependence on the positioning, i.e. angle, of the overhead OR lights. Second, the linearity of the measured intensity and illumination was evaluated by keeping the integration time constant and varying the intensity of the OR lights. The measured intensity collected by all three filters also changes linearly with varying sample emission (Fig. 5c, ${R}^{2}>0.97$). Once background subtraction was performed, all regression lines had slopes of ~ 1 and y-intercepts of ~ 0 as expected (Fig. 5d).

Having characterized and confirmed the linearity of the clinical MIS, seven patients undergoing thyroidectomy were recruited for a human feasibility study. At the surgeons’ discretion, the recurrent laryngeal nerves (RLN) were imaged intraoperatively. The surgeon was asked to outline and point to the nerves in the surgical field (i.e. RLN, motor branch of RLN, and esophageal nerve) during imaging to verify imaging results. The surgeon was blinded to the system display and the images obtained with the MIS. Figure 5e–h and j–m show the white light image, the ${R}_{450/591}$ image, the ${R}_{653/591}$ image, and the combined intensity respectively from two separate patients. Triangles and the surgical forceps point to branches of the recurrent laryngeal nerve. Ratiometric images were combined as previously described. Both ratios significantly reduce the background signal from the surrounding tissue while successfully identifying the indicated nerve branches. As seen in Fig. 5h, however, the entirety of the nerve branch appearing lowest in the image (labeled with three triangles) was not completely highlighted in the overlayed image. Across all seven patients, the signal-to-background ratio (SBR) was substantially higher using ${R}_{450/591}$ than ${R}_{653/591}$ when comparing the signal from nerve to muscle and fat (Fig. 5i, n). Additionally, the SBR of nerve compared to muscle is higher than that of nerve to fat.

## Discussion

The risk of iatrogenic nerve injury is a real and dreadful surgical complication for both surgeons and patients. Intraoperative nerve visualization has the potential to reduce the prevalence of iatrogenic nerve injuries by enabling surgeons to accurately localize and identify nerves. The goal of this work is to demonstrate the efficacy of low-cost DRS for intraoperative nerve detection and visualization. Since surgeons have differing preferences on the implementation, both fiber- and imaging-based approaches were evaluated. Leveraging the biochemical and functional information provided by DRS as well as its label-free nature, a fiber-based DRS study was first performed in an in vivo rat model to identify wavelengths capable of distinguishing nerve from surrounding tissues.

Across the spectral range of 400–800 nm, PCA identified two wavelengths, 450 and 653 nm, responsible for most of the variability seen in the diffuse reflectance spectra of nerve, fat, muscle, and bone (Fig. 1d). A normalizing factor was also identified at 591 nm that contributed minimally to the overall variance in the data. Hyperspectral imaging independently identified similar wavelengths, 452, 712, and 599 nm, using the same statistical analysis (Fig. 2d). While the wavelengths at 450 and 452 nm as well as 591 and 599 nm were practically identical, the 653 and 712 nm wavelengths were not. This disparity is understandable given that the resultant loading coefficients from PCA in Figs. 1d and 2d plateau after ~ 620 nm yielding comparable values for both 653 and 712 nm. More compellingly, in comparing the optical properties at these wavelengths, the difference in absorption and scattering are also relatively small (Fig. 3b, c). However, 653 nm corresponds to a substantial difference in the oxygenated and deoxygenated hemoglobin absorption spectra which is not the case at 712 nm (Fig. 3a). Moreover, ${R}_{653/591}$ and ${R}_{712/591}$ yielded almost identical results (Figs. 2h and S1 respectively) in terms of statistical significance between tissue types. Taken together, 653 nm was chosen as the discriminatory wavelength candidate along with 450 and 591 nm.

The ratios of the diffuse reflectance intensity at the identified wavelengths, ${R}_{450/591}$ and ${R}_{653/591}$, produces statistically distinct populations for each tissue type. In the fiber-based study, the ${R}_{450/591}$ of nerve is significantly less than that of bone, fat, and muscle (Fig. 1e). The difference in ${R}_{450/591}$ between nerve and fat as well as nerve and muscle is also statistically significant using the imaging-based data. Nerve, however, exhibited a higher ${R}_{450/591}$ than the other tissues in the imaging data (Fig. 2e). This difference is also reflected in the results of the respective principal component analysis. Whereas 450 nm is the maximum positive loading coefficient for the fiber-based data (Fig. 1d), 450 nm is the minimum negative loading coefficient according to the results of the imaging-based study (Fig. 2d). Ultimately, this discrepancy is likely due to the spatial differences in the illumination-collection geometries between the fiber- and imaging-based systems. While probe-based systems collect light from the sub-diffuse regime due to their fixed fiber geometry, imaging-based approaches collect more diffuse photons as each pixel is collecting photons from all possible source-detector separation distances^52^. Previous studies have observed that imaging spectra exhibit greater intensity contrast between the 400–600 nm and 600–720 nm ranges than probe spectra^52^. This likely accounts for the reversal in the ${R}_{450/591}$ magnitude between the two systems as well as the discrepancy between the 653 and 712 nm discriminatory wavelengths. Nonetheless, the ${R}_{450/591}$ of nerve remained statistically distinct from the other tissues regardless of which system was employed.

While the selection of the three wavelengths was determined statistically, the statistical analyses were not accepted *prima facie*, and the underlying source of contrast for nerve discrimination was investigated. Examination of the absorption spectra of the rat tissues revealed that 450 nm coincides with an isosbestic point in the absorption of all considered tissues (Fig. 3b). This is also coincident with the isosbestic point in the extinction coefficient spectra of oxygenated and deoxygenated hemoglobin (Fig. 3a). Conversely, the isosbestic point is less evident in the human cadaver absorption spectra (Fig. 3d). It should be noted, however, that the human tissues were frozen prior to conducting reflectance and transmittance measurements. Both the freezing and thawing process can alter the optical properties of the tissue from their in vivo ondition. The deoxygenated state of the rat absorption spectra is likely due to the still living tissue consuming the remaining bound oxygen. In contrast, the human tissue absorption spectra were acquired from frozen specimens. During the thawing process, hemoglobin can bind to oxygen in the air. However, since the tissue is no longer living, the oxygen remains bound to the hemoglobin. Given that the absorption coefficient of the tissues is extremely similar if not equal at 450 nm in human tissues, the source of contrast was hypothesized to stem from the higher degree of scattering in nerves.

Spectra of the reduced scattering coefficient ($\mu_{s}^{\prime }$) confirms this hypothesis. Nerve tissue has a substantially higher $\mu_{s}^{\prime }$ than every other tissue except tendon not only at 450 nm but also across the entire spectral range (Fig. 3c). This trend also holds for $\mu_{s}^{\prime }$ calculated from the human cadaver tissues (Fig. 3e). This then explains why nerves have the highest overall reflectance regardless of which system is used to collect the spectra. We hypothesize that the higher $\mu_{s}^{\prime }$ of nerve is likely due to the high lipid content of nerves that stems from their myelin sheaths as well as the various collagenous sheaths surrounding them (i.e. the epineurium, perineurium, and endoneurium). The normalizing wavelength at 591 nm also corresponded to an isosbestic in the hemoglobin spectra (Fig. 3a). The absorption coefficients of all the rat tissues studied were similar at 591 nm (Fig. 3b). While 591 nm is an ideal candidate for a normalizing factor from an absorption perspective, it should be noted that 591 nm is still affected by the varying degrees of scattering of the different tissues (Fig. 3c). In addition to 450 nm, 653 nm was also identified as a candidate for nerve discrimination.

The ${R}_{653/591}$ of nerve is statistically significant from that of bone and fat but not muscle based on spectra acquired with the fiber-probe (Fig. 1f). Using the hyperspectral imaging data, the ${R}_{653/591}$ of nerve was also statistically distinct from that of ${R}_{653/591}$ of muscle but that not fat (Fig. 2h). Since 653 nm corresponds to the minimum negative and the maximum positive PCA loading coefficient from the fiber-probe and imaging system data respectively, the ${R}_{653/591}$ of nerve is higher for the probe-based system (Figs. 1d, 2d). Interestingly, the difference in ${R}_{653/591}$ between nerve and fat is statistically significant for the fiber-based DRS but not for the imaging-based. To this point, the spectra of fat acquired with the hyperspectral camera clearly overlap with those from nerve particularly after ~ 600 nm (Fig. 2b). In the ${R}_{653/591}$ image, the signal from fat is more pronounced than in the ${R}_{450/591}$ image (Fig. 2m and n respectively). Whereas the ${R}_{653/591}$ of muscle was distinct from nerve using the fiber-probe, it is not when calculated using the imaging data. This is again likely due to differences in illumination-collection geometries between the systems as described above. The results of the fiber-based system, however, align well with the insights gained from the optical property measurements.

As can be observed from the hemoglobin extinction coefficient spectra, 653 nm coincides to a considerable separation between the oxygenated and deoxygenated spectra and is adjacent to wavelengths commonly utilized for pulse oximetry (Fig. 3a). This is important to note since this is not necessarily reflected in the rat tissue absorption spectra which were acquired using ex vivo tissue specimens that contain varying concentrations of both oxygenated and deoxygenated hemoglobin. For instance, most tissues appear to be deoxygenated except for tendon and fat (Fig. 3b). Despite the fact that the optical properties of ex vivo tissue specimens do not accurately represent those of in vivo tissue especially when considering blood related signatures, the differences in absorption coefficients between the tissues are still evident at 653 nm in the rat tissue and to some extent in the thawed human cadaver tissues (Fig. 3b and d respectively). Again, the freezing and thawing of the human tissue specimens as well as its ex vivo state likely altered the oxygenation state of the tissue modulating the effects of hemoglobin in the ex vivo tissues from their true in vivo state. The ${R}_{653/591}$ of the considered tissues, however, also offers evidence to support the source of contrast at 653 nm is based on oxygenation. In the fiber-based rat and human studies, both nerve and muscle have a higher ${R}_{653/591}$ than many of the other tissues (Figs. 1e, 4c). Based on physiology, both muscle and nerve require a greater blood and energy supply to function than tissues like fat or tendon. With a higher energy demand and thus oxygen consumption, more oxygenated hemoglobin may be present in these tissues reducing the amount of absorbed light at 653 nm resulting in a higher ${R}_{653/591}$. This may also explain why the freshly extracted rat muscle and nerve tissues are deoxygenated as the tissues may have continued to consume the remaining oxygen at a higher rate while they were still viable. While scattering cannot be discounted, taken together, the data suggests the source of contrast at 653 nm is likely due to differences in oxygenated and deoxygenated hemoglobin content and may be detecting a difference in bulk oxygenation or total hemoglobin content of nerve tissue. This implies that the discriminatory power of ${R}_{653/591}$ may be contingent on tissue perfusion and/or oxygenation status.

Despite the noted differences in the trends between fiber- and imaging-based systems, the results from each system are promising. Linear discriminant analysis (LDA) performed on the fiber-based data using both ratios derived from the three key wavelengths successfully separated nerve from fat, muscle, and bone from rats in vivo with an accuracy of 92% (Fig. 1g and h). Since the aim of the imaging-based study is to enhance nerve visualization, simple image processing was used to combine the ${R}_{450/591}$ and ${R}_{653/591}$ images to highlight the distribution of nerves in the surgical field (Fig. 2n). False positive results from the LDA are primarily misclassifications of fat and tendon. Similarly, in the imaging data, areas in the surgical field misidentified as nerve consisted of fat deposits. Given the efficacious performance of the ratiometric approach in the rat model for both fiber- and imaging-based data, human feasibility studies were conducted using the same fiber-based DRS system and the custom clinical MIS.

A fiber-based DRS human feasibility was conducted in 6 patients undergoing thyroidectomy. The trends for ${R}_{450/591}$ and ${R}_{653/591}$ in rats are consistent with those in the clinical human study. The ${R}_{450/591}$ of human nerve is lower than the other tissues and significantly lower than that of muscle, tendon, and thyroid gland (Fig. 4b). The ${R}_{653/591}$ of human nerve and muscle is higher than that of the other tissues and significantly so for tendon, fat, and thyroid gland (Fig. 4c). Parathyroid glands overall did not have statistically distinct ${R}_{450/591}$ and ${R}_{653/591}$ from nerve. This could possibly be due to higher scattering in parathyroid glands (lower ${R}_{450/591}$) and a greater vascular density (higher ${R}_{653/591}$) due to their small size. Since the measurements included lymph node, parathyroid, and thyroid tissue in addition to nerve, muscle, and fat, a new LDA model was trained using the ratios calculated from human spectra (Fig. 4d). The human LDA model has a comparable accuracy (91%) to the one trained on the rat data (Fig. 4e). Again, fat accounted for most of the false positives. The 80% sensitivity of the human LDA model is primarily due to the small sample size of the study. We anticipate that as the number of patients recruited for the study is increased, the performance of the technique in humans will map that observed in rats in vivo.

Since performing measurements with OR lights on is crucial to maintaining proper surgical workflow, the clinical MIS was designed and characterized to exploit the existing OR lights as the illumination source. After establishing the linearity of the system (${R}^{2}>0.97$) with respect to measured intensity, excitation intensity, and integration time, variable integration times could be exploited to ensure adequate signal collection at the three wavelengths (Fig. 5d). The major drawback of utilizing the OR lights as the excitation source is the presence of specular reflections which are noticeable in white light, ratioed, and combined images (Fig. 5e–h and 5j-m). Nonetheless, ${R}_{450/591}$ and ${R}_{653/591}$ images successfully suppress the background and highlight all nerve branches in the surgical field. Aside from specular reflections, the combined ${R}_{450/591}$ and ${R}_{653/591}$ images also clearly delineate the distribution of nerves in the surgical field. The ${R}_{450/591}$ images also provide a substantially higher signal-to-background ratios (SBR) for nerve tissue than the ${R}_{653/591}$ images (Fig. 5i). The SBR of nerve to fat is also lower than that of nerve to muscle because nerve is optically more similar to fat than muscle as seen in the diffuse reflectance spectra and optical properties measurements. While the evidence presented here establishes the clinical potential of DRS for intraoperative nerve detection and visualization, clear improvements can be made.

Adding polarization imaging capabilities could further improve the label-free discriminatory power of the current approach. As fat accounted for many of the false positives in the fiber-based studies and reduced the SBR in the imaging data, incorporating polarization could further help distinguish fat from nerve based on the intrinsic depolarization and birefringence of fat and nerve respectively^36–39^. Moreover, orthogonal polarization filtering would also significantly reduce the contributions of specular reflections in clinical imaging systems as seen with the animal imaging pictures (Fig. 2k–n). The addition of crossed polarizers on the excitation and collection ends of existing laparoscopic, endoscopic, and microscopic systems could be accomplished with simple modifications. Linear polarizers could also be mounted to OR lights.

Wavelengths further into the infrared pertaining to lipid and water absorption might also provide additional discriminatory power. Moreover, the depth of detection was also quantified for the ratios and ranged from 311 to 383 µm under fat or muscle for the $1/e$ signal attenuation (Fig. 2f–g and i–j). This limits the nerve detection and visualization superficial layers. Utilizing wavelengths further into the infrared region,however, could improve the pentration depth while provide lipid and water information.

Further modifications to the imaging systems can also easily be made to enable real-time imaging. The images presented here were not acquired in real-time primarily due to the long transition time needed to switch between spectral filters. The spectral power output of the OR lights used in this study, however, made it possible to acquire images at integrations times < 3 ms. Exchanging the motorized filter wheel for on-chip filters, acousto-optic tunable filter, or liquid crystal tunable filter, however, would dramatically decrease total image acquisition times. Real-time imaging could also eliminate the need for motion correction of the acquired images. Likewise, the fiber-based DRS system could also be optimized for smoother integration into the OR.

Spectra collected using the probe were acquired with the OR and/or room lights off which is not conducive to the surgical workflow. Various methods, such as heterodyne detection, could be employed to allow measurements to be made while keeping the OR lights on. The system itself could also be simplified and made more cost-effective by replacing the spectrometer with individual spectral channels. While various modifications can be made to the probe-based as well as imaging-based DRS systems to enhance performance in the future, results presented in this work indicate the potential of DRS for intraoperative nerve visualization and therefore minimizing the incidence of iatrogenic nerve injury. It should also be noted that there are current surgical applications that could benefit from superifical nerve detection such as procedures involving nerve reconstruction and establishing proper nerve-neuroprosthetic interfaces.

## Conclusion

In this paper, the potential of a label-free, non-invasive ratiometric approach based on DRS for intraoperative nerve detection and visualization is presented. Both probe-based and imaging-based approaches were evaluated in an in vivo animal model and validated in human feasibility studies. With the ability to identify and localize nerves, this tool has the potential to reduce the prevalence of iatrogenic nerve injuries across a wide array of surgical procedures as well as reduce the subsequent costs and damages they incur.

## Materials and methods

### Animal preparation

All experiments were conducted at the Vanderbilt Biophotonics Center in adherence to protocols approved by the Vanderbilt Institution of Animal Care and Use Committee (IACUC) and are reported in accordance with ARRIVE guidelines where applicable. All methods were performed according to the relevant ethical guidelines and regulations as approved by the Vanderbilt IACUC. Tissue spectra were collected in vivo from adult male and female Sprague–Dawley rats ($n=33$, 250–300 g) following a sciatic nerve preparation. Detailed surgical procedure has been described previously^53^. Briefly, animals were anesthetized with isoflurane before a ~ 3 cm incision was made laterally from the gluteus muscles to the popliteal region. A split-muscle technique was then used to expose the sciatic nerve. Rats were euthanized following experimental completion in accordance with the IACUC approved protocol before tissue samples were harvested for optical property measurements.

### Patient recruitment

All human studies were conducted in accordance with the Declaration of Helsinki and its amendments as well as approved by the Vanderbilt University Medical Center (VUMC) Institutional Review Board (IRB) (IRB#: 070795 and 181544). Patients undergoing thyroidectomy were recruited at VUMC and written informed consent was obtained from each patient ($n=13$) prior to participation. Spectra or images were acquired at the discretion of the surgeon over the course of the surgery. The surgeon was blinded to the acquired data until the completion of the procedure. Visual inspection of the surgeons served as the gold standard in identifying tissues.

### Fiber-based diffuse reflectance spectroscopy and protocol

Fiber-based diffuse reflectance spectra were collected using a computer controlled portable spectroscopy system depicted in Fig. 1a. A 150 W tungsten-halogen lamp (Ocean Optics, Dunedin, FL) was used as a broadband white light illumination source providing emission from 400-800 nm with an average output of 0.56 mW at the probe face. Light from the lamp was coupled into a commercially constructed, sterilizable, reusable, custom designed fiberoptic probe (Visionex, Inc., Atlanta, GA). The fiber-probe consists of seven 300 µm fibers arranged in a six-around-one configuration with two illumination fibers on opposite sides of the central fiber (Fig. 1a). The remaining five collection fibers delivers diffuse reflectance signal to a VIS–NIR spectrometer (S2000-FL; Ocean Optics, Dunedin, FL). The spectrometer was routinely wavelength calibrated using HgAr and NeAr lamps and determined to have a spectral resolution of 2.4 nm. Customized software developed in LabView was used to collect all spectral data in this study.

The fiber-probe was disinfected with 10% bleach solution between measurements from each animal. All spectra were collected in vivo with the room lights off. Once the tip of the fiber-probe was placed in gentle contact with the target tissue, spectra were collected using a 100 ms acquisition time. Tissue spectra were collected from three separate locations of the target tissue. At each location, six spectra were taken from each location and averaged. All tissue spectra were collected while the probe remained in contact with the tissue. Background spectra were then taken with the halogen lamp turned off. Reference spectra were collected with the lights off after each study from a 99% diffuse reflectance standard (Labsphere, North Sutton, NH) to account for any variations in delivered light output.

Prior to each human study, the fiber-probe was sterilized by low temperature sterilization (V-PRO maX; Steris, Mentor, OH). Spectra were acquired intraoperatively with room lights and overhead OR lights turned off. The fiber-probe placement was performed by the surgeon with the probe in gentle contact with the tissue using a 100 ms acquisition time. For a given tissue, three tissue spectra were taken from two locations to minimize added surgical time. Tissue identification was performed by the participating surgeon and was considered the “gold standard”. Background and reference spectra were then taken as previously described.

### Imaging-based diffuse reflectance spectroscopy

For animal experiments, a Fabry-Pérot interferometer-based, hyperspectral camera (VNIR; HinaLea, Emeryville, California) was used to image rat sciatic nerve preparations in vivo. A liquid light guide (Steiner and Martins, Miami, Florida) coupled to a 200 W halogen lamp (Luxtec, West Boylston, Massachusetts) was used as the excitation source for imaging-based DRS. The hyperspectral camera was wavelength calibrated with NeAr and HgAr lamps and found to have a spectral resolution of 10.3 nm. The liquid light guide was angled obliquely to the surgical field, and the camera was positioned vertically above the sample to reduce specular reflections. Additionally, orthogonally oriented linear polarizers are placed at the output of the liquid light guide and before the collection optics of the hyperspectral camera to further eliminate specular reflections. Background spectra were collected with the halogen lamp off. Animals were imaged with room lights off over a spectral range of 400–800 nm at an integration time of 1 s to ensure adequate SNR (2/3 of the dynamic range) and to prevent saturation. A 99% diffuse reflectance standard was imaged after experiments as a reference. Areas corresponding to each tissue type were manually segmented and averaged to create an average spectrum for each tissue type per experiment. The spatial resolution of the system was also characterized by imaging a 1951 U.S. Air Force resolution target (R3L1S4N; Thorlabs Inc., Newton, NJ). Using a peak-to-valley ratio equal to $\sqrt{2}$ as the threshold^54^, group 2, element 1 was resolved equating to a resolution of 125 µm^54^.

The depth of detection using the hyperspectral imaging system was determined using ex vivo rat sciatic nerve specimens overlaid with varying thicknesses of fat and muscle. Uncured bacon fat and muscle were sliced using a microtome to thickness of 200 and 300 µm respectively. Excised nerve samples were placed on a glass slide and imaged under 0, 200, 400, 600, 1000 µm of fat and 0, 300, 600, and 900 µm of muscle. Images were then ratioed at the identified wavelengths. To account for background signal of the surrounding tissue (i.e., fat or muscle), regions of nerve were divided by a region of equal area of fat or muscle to obtain a signal-to-background ratio. After normalizing to the signal at 0 µm, data was fitted to an exponential decay function using a least-squares fitting approach. The $1/e$ attenuation of the ratio’s signal was used to quantify the depth of detection.

### Spectral processing and analysis

Before spectral analyses, all raw spectra were processed to account for different sources of variation. The background spectrum ($${B}_{i}(\lambda$$)) for the $$i$$-th experiment was subtracted from the corresponding tissue spectrum ($${T}_{i}(\lambda$$)) and reference spectrum ($${R}_{i}(\lambda$$)). Since the reference spectrum was collected using a 99% diffuse reflectance standard, a correction factor ($$c_{99} = {\raise0.7ex\hbox{${100}$} \!\mathord{\left/ {\vphantom {{100} {99}}}\right.\kern-0pt} \!\lower0.7ex\hbox{${99}$}}$$) was also applied to the reference spectrum such that the processed spectrum ($${DR}_{i}(\lambda$$)) took the form:

$$DR_{i} \left( \lambda \right) = \frac{{T_{i} \left( \lambda \right) - B_{i} \left( \lambda \right)}}{{c_{99} *R_{i} \left( \lambda \right) - B_{i} \left( \lambda \right)}}$$

Blood dominated spectra were not considered in subsequent analyses. Blood dominated spectra were defined as spectra having an intensity at 562 nm, a center point between oxygenated and deoxygenated hemoglobin absorption peaks, that was three standard deviations below the mean of the appropriate tissue type.

To identify optimal wavelengths for nerve discrimination, processed spectra were normalized to the area under the curve from 400 to 800 nm before performing principal component analysis. Resultant loading plots from the first and second principal component were then used to identify wavelengths responsible for the most variance and a normalization wavelength contributing minimally to the variance. Once the wavelengths were selected, ratios of the discriminatory wavelengths to the normalization wavelength were calculated. Linear discriminate analysis was then performed with k-fold cross validation ($k=5$) to determine sensitivity and specificity. The ground truth for tissue classification was determined by the experienced surgeon’s or animal personnel’s identification. Nerve tissue was further confirmed in vivo via electrical stimulation and electromyography recordings or muscle twitches in rats. All processing and analyses were performed in MATLAB (Mathworks, Natick, Massachusetts).

### Image processing

Image processing was designed to replicate spectral analysis. Images taken at the discriminatory and normalization wavelengths were used to create ratio images. A 3-class Reddi thresholding algorithm was then applied to ratiometric images to isolate pixels corresponding to nerve tissue from those of other tissues and specular reflections^55^. The two ratioed images were then summed and converted to binary images. To eliminate any residual specular reflections, thresholded images were eroded and then dilated. To create a single overlay highlighting nerve tissue in the field of view, the binarized and white light images were summed with the binarized overlay set to be saturated in the green channel. Regions corresponding to skin, fur or surgical tools were manually excluded from analyses. If motion correction was needed, simple linear transformations were applied manually. Image processing was performed in ImageJ (National Institute of Health, Bethesda, Maryland). Image contrast was adjusted to enhance visualization.

### Tissue preparation and optical properties measurement

Optical properties were measured using freshly excised rat sciatic nerve, abdominal muscle, subcutaneous fat, and tail tendon harvested immediately after surgery. Human tissues obtained through the Vanderbilt Cooperative Human Tissue Network were excised from thawed cadaver arms and included ulnar and radial nerves, subcutaneous fat, extensor and flexor muscles, and flexor tendons. Rat tissues were stored at 4 °C in airtight containers, along with a damp physiological saline wipe, before making measurements. Human tissues, which were previously frozen before harvesting, were stored at − 80 °C. All measurements were made after tissues had thawed to room temperature. Tissues were then placed between two glass slides fitted with spacers (ranging in thickness from 0.23 to 0.73) to avoid tissue compression and ensure constant sample thickness.

Total reflectance and transmittance were measured using a dual-beam, single integrating sphere spectrophotometer (Cary 7000 Spectrophotometer, Agilent, Santa Clara, California) from 400 to 800 nm in 4 nm steps. To ensure sufficient collection of radially scattered photons, the beam size at the sample was maintained at ~ 2 mm^2^ so that the spot size of the beam remained < 5% of the total sample area. In order to avoid first-order sphere corrections, reflectance and transmittance from measured specimens were normalized to the reference beam incident upon a 99% diffuse reflectance standard. Losses originating from air-glass specular reflections and/or glass slide absorption of the sample holder were also corrected for by normalizing reflectance sample spectra to a reference spectrum acquired with a single glass slide placed in front of the 99% DR standard on the reflectance port and transmittance sample spectra to reference spectra acquired with a single glass slide in the transmittance port. Normalized total reflectance and transmittance spectra of specimens were then used to iteratively determine the absorption and reduced scattering coefficient via the inverse adding doubling (IAD) method^50^. Prior to tissue characterization, 1.1 µm polystyrene spheres (5100A; Fisher Scientific, Waltham, Massachusetts), whose scattering properties are well described by Mie theory, at a concentration of 0.0054 v/v in deionized water were used to validate reduced scattering coefficient calculations by fitting the results to the power-law Mie-Rayleigh scattering equation (Fig S2a)^56^:

$$\mu_{s}^{\prime } \left( \lambda \right) = \mu_{0} \left[ {f_{Ray} \left( {\frac{{\lambda^{ - 4} }}{{500\;{\text{nm}}}}} \right) + \left( {1 - f_{Ray} } \right)\left( {\frac{{\lambda^{{ - b_{Mie} }} }}{{500\;{\text{nm}}}}} \right)} \right]$$

where ${\mu }_{0}$ is the scattering coefficient at 500 nm, ${f}_{Ray}$ is the Rayleigh fraction, $\lambda$ is the wavelength, and ${b}_{Mie}$ is the “Mie power.” All fittings were performed using linear least absolute residuals. Absorption coefficient results were validated using 10.8 mmol/L concentration of methylene blue, a well characterized chromophore in the visible range, in deionized water at 293 K (Millipore Sigma, Burlington, Massachusetts) (Fig. S2b)^51^.

### Clinical spectral imaging characterization and protocol

A clinical multispectral imaging system (MIS) was developed to image nerves during thyroidectomies at the identified wavelengths (Fig. 5a). The entire system was mounted to an articulated arm fixed to a mobile cart. The articulated arm was fitted with an insertion site for sterile handles to be attached that allowed surgeons to position the camera intraoperatively. The imaging system consists of a CMOS camera (acA1300-60 gmNIR; Basler AG, Ahrensburg, Germany), imaging lens system (Navitar 50 mm F/2.8; Navitar, Woburn, MA), a motorized filter wheel (FW102C; Thorlabs, Inc., Newton, NJ), and a focus tunable lens (EL-16-40-TC-VIS-5D-M27; Optotune, Dietikon, Switzerland). Two 5 mW 660 nm laser pointers (DigiKey, Thief River Falls, MN) were angled such that they colocalized in the center of the field of view at a distance of ~ 50 cm from the imaging system so that it remained outside of the sterile zone and allowed the surgeon to accurately position the camera. The focus tunable lens was used to compensate for any variations in the distance between the patient’s surgical site and camera so that the images remained in focus. The lens set was designed to provide a ~ 3 × 3 cm field of view and a 100 µm spatial resolution. Three filters centered at the identified wavelengths are installed in the filter wheel: 448 ± 10 nm band pass filter (FF01-448/20-25; Semrock, Rochester, NY), 590 ± 6 nm band pass filter (FF01-591/6-25; Semrock, Rochester, NY), 655 ± 8 nm band pass filter (FF01-655/15-25; Semrock, Rochester, NY). The clinical MIS is designed to utilize the operating room lights as the excitation source. The entire system is controlled in MATLAB via a custom graphic user interface (GUI) on a laptop computer. The spatial resolution of the system was characterized by imaging a 1951 U.S. Air Force resolution target. Using a peak-to-valley ratio equal to $\sqrt{2}$ as the threshold, group 2, element 2 was resolved equating to a resolution of 111 µm.

System linearity was assessed by imaging a 99% diffuse reflectance standard in a mock OR equipped with the same surgical OR lights used at VUMC. To determine the linearity of measured intensity as a function of integration time, the standard was placed on the operating table and illuminated by the OR lights held at a constant intensity while being imaged at varying integration times (10 µs–512 ms). This was repeated multiple times with the OR lights at various angles (0–60°) to also determine if the linearity of measured intensity had any angular dependence. Background images with the OR lights turned off were also acquired at each integration time and subtracted from the corresponding image with the OR lights on. The mean intensity across the standard was then calculated for each background subtracted image. Integration times and mean intensities were normalized to their maximum values. A linear least squares fitting was then performed on the normalized data. Similar testing and analysis were performed to determine the linearity of measured intensity with respect to excitation intensity. For this test, integration time was held constant at 2 ms while the excitation intensity of the OR lights was varied using the control panel. This characterization was performed for each of the three spectral filters.

During surgery, white light images and spectral images were acquired with the OR lights on. The surgeon was asked to trace and point to the RLN nerve during imaging which served as the ground truth for identification. In order to acquire adequate signal (at least ½ the dynamic range) and account for the spectral sensitivity of the system, individual integration times for each of the filters was varied such that spectral images had an average intensity equal to 1/2 the dynamic range of the camera (2048 counts) during intraoperative imaging ($${t}_{acquired})$$. Since images were acquired within the linear response region of the camera and at different integration times, a correction factor was calculated $$\left( {a = \frac{{t_{acquired} }}{{{\text{max}}\left( {t_{acquired} } \right)}}} \right)$$ normalizing each of the three images to the one with the longest integration time. The correction factor was then applied to the whole image to correct for different integration times and ensure proportionate signal at each identified wavelength.

## Supplementary Information

Supplementary Information.

## Data Availability

The datasets generated and analyzed during the current study are available from the corresponding author on reasonable request.

## References

[1] Stanford JL, Feng Z, Hamilton AS, Gilliland FD, Stephenson RA, Eley JW (2000). Urinary and sexual function after radical prostatectomy for clinically localized prostate cancer. JAMA.

[2] Wang K, Yee C, Tam S, Drost L, Chan S, Zaki P (2018). Prevalence of pain in patients with breast cancer post-treatment: A systematic review. Breast.

[3] Andersen KG, Kehlet H (2011). Persistent pain after breast cancer treatment: A critical review of risk factors and strategies for prevention. J. Pain.

[4] Kaur N, Kumar A, Saxena AK, Gupta A, Grover RK (2018). Postmastectomy chronic pain in breast cancer survivors: An exploratory study on prevalence, characteristics, risk factors, and impact on quality of life. Indian J. Surg..

[5] Antoniadis G, Kretschmer T, Pedro MT, König RW, Heinen CPG, Richter H-P (2014). Iatrogenic nerve injuries: Prevalence, diagnosis and treatment. Dtsch. Arztebl. Int..

[6] Michael Henry B, Pe PA, Sanna B, Vikse J, Sanna S, Saganiak K (2017). The anastomoses of the recurrent laryngeal nerve in the larynx: A meta-analysis and systematic review. J. Voice.

[7] Henry BM, Graves MJ, Vikse J, Sanna B, Pękala PA, Walocha JA (2017). The current state of intermittent intraoperative neural monitoring for prevention of recurrent laryngeal nerve injury during thyroidectomy: A PRISMA-compliant systematic review of overlapping meta-analyses. Langenbecks Arch. Surg..

[8] Hall MJ, DeFrances CJ, Williams SN, Golosinskiy A, Schwartzman A (2010). National hospital discharge survey: 2007 summary. Natl. Health Stat. Rep..

[9] Defrances CJ, Lucas CA, Buie VC, Golosinskiy A. *National Health Statistics Reports Number 5* (2006). 18841653

[10] Defrances, C. J., Hall, M. J. *Vital and Health Statistics, Advance Data 385*, Vol. 385 (2005).

[11] Delank KS, Delank HW, König DP, Popken F, Furderer S, Eysel P (2005). Iatrogenic paraplegia in spinal surgery. Arch. Orthop. Trauma Surg..

[12] Kaylie DM, Gilbert E, Horgan MA, Delashaw JB, McMenomey SO (2001). Acoustic neuroma surgery outcomes. Otol. Neurotol..

[13] Abadin SS, Kaplan EL, Angelos P (2010). Malpractice litigation after thyroid surgery: The role of recurrent laryngeal nerve injuries, 1989–2009. Surgery.

[14] Barczyński M, Konturek A, Pragacz K, Papier A, Stopa M, Nowak W (2014). Intraoperative nerve monitoring can reduce prevalence of recurrent laryngeal nerve injury in thyroid reoperations: Results of a retrospective cohort study. World J. Surg..

[15] Campbell WW (2008). Evaluation and management of peripheral nerve injury. Clin. Neurophysiol..

[16] Kehlet H, Jensen TS, Woolf CJ (2006). Persistent postsurgical pain: Risk factors and prevention. Lancet.

[17] Morris LGT, Ziff DJS, DeLacure MD (2008). Malpractice litigation after surgical injury of the spinal accessory nerve. Arch. Otolaryngol. Neck Surg..

[18] Wilson TJ, Yang LJS (2018). Peripheral Nerve Surgery.

[19] Grayev A, Reeder S, Hanna A (2016). Use of chemical shift encoded magnetic resonance imaging (CSE-MRI) for high resolution fat-suppressed imaging of the brachial and lumbosacral plexuses. Eur. J. Radiol..

[20] Manoliu A, Ho M, Nanz D, Piccirelli M, Dappa E, Klarhöfer M (2016). Diffusion tensor imaging of lumbar nerve roots. Invest. Radiol..

[21] Filler AG, Kliot M, Howe FA, Hayes CE, Saunders DE, Goodkin R (1996). Application of magnetic resonance neurography in the evaluation of patients with peripheral nerve pathology. J. Neurosurg..

[22] Haldeman CL, Baggott CD, Hanna AS (2015). Intraoperative ultrasound-assisted peripheral nerve surgery. Neurosurg. Focus.

[23] Lee FL, Singh H, Nazarian LN, Ratliff JK (2011). High-resolution ultrasonography in the diagnosis and intraoperative management of peripheral nerve lesions. J. Neurosurg..

[24] Matthews TP, Zhang C, Yao D-K, Maslov K, Wang LV (2014). Label-free photoacoustic microscopy of peripheral nerves. J. Biomed. Opt..

[25] Horiguchi A, Tsujita K, Irisawa K, Kasamatsu T, Hirota K, Kawaguchi M (2016). A pilot study of photoacoustic imaging system for improved real-time visualization of neurovascular bundle during radical prostatectomy. Prostate.

[26] Finke M, Kantelhardt S, Schlaefer A, Bruder R, Lankenau E, Giese A (2012). Automatic scanning of large tissue areas in neurosurgery using optical coherence tomography. Int. J. Med. Robot. Comput. Assist. Surg..

[27] Islam MS, Oliveira MC, Wang Y, Henry FP, Randolph MA, Park BH (2012). Extracting structural features of rat sciatic nerve using polarization-sensitive spectral domain optical coherence tomography. J. Biomed. Opt..

[28] Dip F, Bregoli P, Falco J, White KP, Rosenthal RJ (2022). Nerve autofluorescence in near-ultraviolet light markedly enhances nerve visualization in vivo. Surg. Endosc..

[29] Dip F, Rosenthal D, Socolovsky M, Falco J, De la Fuente M, White KP (2021). Nerve autofluorescence under near-ultraviolet light: Cutting-edge technology for intra-operative neural tissue visualization in 17 patients. Surg. Endosc..

[30] Wang LG, Barth CW, Kitts CH, Mebrat MD, Montaño AR, House BJ (2020). Near-infrared nerve-binding fluorophores for buried nerve tissue imaging. Sci. Transl. Med..

[31] Barth CW, Gibbs SL (2017). Direct administration of nerve-specific contrast to improve nerve sparing radical prostatectomy. Theranostics.

[32] Whitney MA, Crisp JL, Nguyen LT, Friedman B, Gross LA, Steinbach P (2011). Fluorescent peptides highlight peripheral nerves during surgery in mice. Nat. Biotechnol..

[33] Huff TB, Cheng J-X (2007). In vivo coherent anti-Stokes Raman scattering imaging of sciatic nerve tissue. J. Microsc..

[34] Farrar MJ, Wise FW, Fetcho JR, Schaffer CB (2011). In vivo imaging of myelin in the vertebrate central nervous system using third harmonic generation microscopy. Biophys. J..

[35] Yadav R, Mukherjee S, Hermen M, Tan G, Maxfield FR, Webb WW (2009). Multiphoton microscopy of prostate and periprostatic neural tissue: A promising imaging technique for improving nerve-sparing prostatectomy. J. Endourol..

[36] Chin KWTK, Engelsman AF, Chin PTK, Meijer SL, Strackee SD, Oostra RJ (2017). Evaluation of collimated polarized light imaging for real-time intraoperative selective nerve identification in the human hand. Biomed. Opt. Express.

[37] Chin, K. W. T. K., Meijerink, A., Chin, P. T. K. Interventional nerve visualization via the intrinsic anisotropic optical properties of the nerves.

[38] Cha J, Broch A, Mudge S, Kim K, Namgoong J-M, Oh E (2018). Real-time, label-free, intraoperative visualization of peripheral nerves and micro-vasculatures using multimodal optical imaging techniques. Biomed. Opt. Express.

[39] Ning B, Kim WW, Katz I, Park CH, Sandler AD, Cha J (2021). Improved nerve visualization in head and neck surgery using mueller polarimetric imaging: Preclinical feasibility study in a swine model. Lasers Surg. Med..

[40] Bigio IJ, Mourant JR (1997). Ultraviolet and visible spectroscopies for tissue diagnostics: Fluorescence spectroscopy and elastic-scattering spectroscopy. Phys. Med. Biol..

[41] Schols RM, ter Laan M, Stassen LPS, Bouvy ND, Amelink A, Wieringa FP (2014). Differentiation between nerve and adipose tissue using wide-band (350–1830 nm) in vivo diffuse reflectance spectroscopy. Lasers Surg. Med..

[42] Langhout GC, Kuhlmann KFD, Schreuder P, Bydlon T, Smeele LE, van den Brekel MWM (2018). In vivo nerve identification in head and neck surgery using diffuse reflectance spectroscopy. Laryngoscope Investig. Otolaryngol..

[43] Stelzle F, Knipfer C, Bergauer B, Rohde M, Adler W, Tangermann-Gerk K (2014). Optical nerve identification in head and neck surgery after Er:YAG laser ablation. Lasers Med. Sci..

[44] Desjardins AE, van der Voort M, Roggeveen S, Lucassen G, Bierhoff W, Hendriks BHW (2011). Needle stylet with integrated optical fibers for spectroscopic contrast during peripheral nerve blocks. J. Biomed. Opt..

[45] Stelzle F, Terwey I, Knipfer C, Adler W, Tangermann-Gerk K, Nkenke E (2012). The impact of laser ablation on optical soft tissue differentiation for tissue specific laser surgery-an experimental ex vivo study. J. Transl. Med..

[46] Stelzle F, Adler W, Zam A, Tangermann-Gerk K, Knipfer C, Douplik A (2012). In vivo optical tissue differentiation by diffuse reflectance spectroscopy. Surg. Innov..

[47] Langhout GC, Kuhlmann KFD, Wouters MWJM, van der Hage JA, van Coevorden F, Müller M (2018). Nerve detection during surgery: Optical spectroscopy for peripheral nerve localization. Lasers Med. Sci..

[48] Hendriks BHW, Balthasar AJR, Lucassen GW, van der Voort M, Mueller M, Pully VV (2015). Nerve detection with optical spectroscopy for regional anesthesia procedures. J. Transl. Med..

[49] Prahl, S., Jacques, S. L., Gratzer, W. B., Kollias, N. *Tabulated Molar Extinction Coefficient for Hemoglobin in Water* (2020). URL: https://omlc.org/spectra/hemoglobin/summary.html

[50] Prahl SA, van Gemert MJC, Welch AJ (1993). Determining the optical properties of turbid media by using the adding–doubling method. Appl. Opt..

[51] Fernandez-Perez A, Marban G (2020). Visible light spectroscopic analysis of methylene blue in water; what comes after dimer?. ACS Omega.

[52] Gebhart SC, Majumder SK, Mahadevan-Jansen A (2007). Comparison of spectral variation from spectroscopy to spectral imaging. Appl. Opt..

[53] Throckmorton G, Cayce J, Ricks Z, Adams WR, Jansen ED, Mahadevan-Jansen A (2021). Identifying optimal parameters for infrared neural stimulation in the peripheral nervous system. Neurophotonics.

[54] Gebhart SC, Thompson RC, Mahadevan-Jansen A (2007). Liquid-crystal tunable filter spectral imaging for brain tumor demarcation. Appl. Opt..

[55] Reddi SS, Rudin SF, Keshavan HR (1984). An optimal multiple threshold scheme for image segmentation. IEEE Trans. Syst. Man Cybern..

[56] Jacques SL (2013). Optical properties of biological tissues: A review. Phys. Med. Biol..

