# Supplementary material for: Label-free intraoperative nerve detection and visualization using ratiometric diffuse reflectance spectroscopy
Source: Sci Rep. 2023 May 10;13:7599. doi: 10.1038/s41598-023-34054-6 (PMC10172349; doi:10.1038/s41598-023-34054-6)
Supplement: Supplementary file 1 — Supplementary Information. [file 41598_2023_34054_MOESM1_ESM.pdf]

SUPPLEMENTAL FIGURES

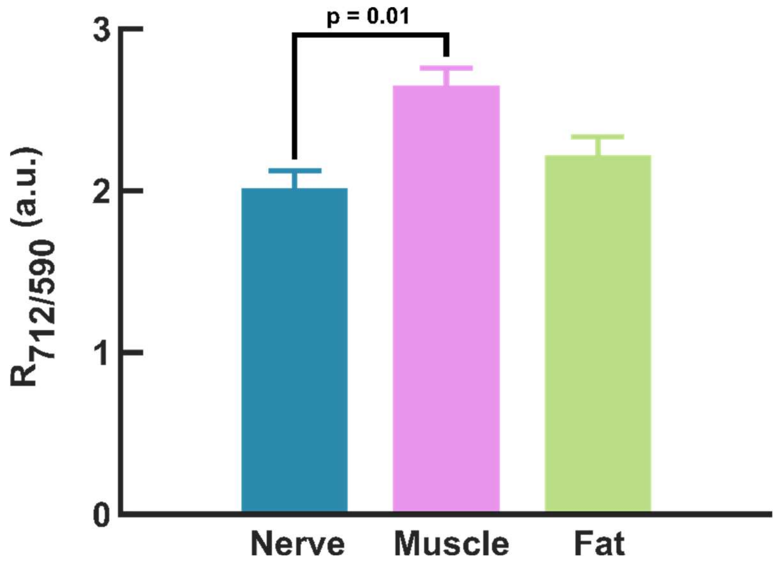

Figure S1. $R_{712/590}$ of rat nerve, muscle, and fat from hyperspectral imaging. Statistical tests for determining significance and reported p-values were calculated using a one-way analysis variance (ANOVA) followed by a Tukey's test. $n = 12$ rats.

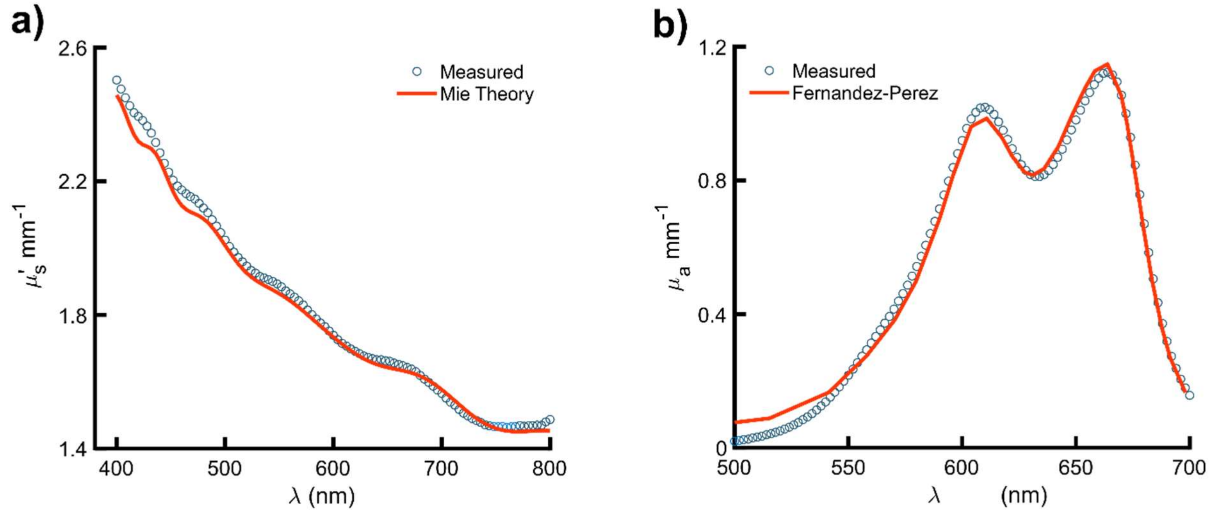

Figure S2. Validation of optical property measurements. (**a**) Measured reduced scattering coefficient of 1.1 µm polystyrene spheres at a concentration of 0.0054 v/v in deionized water (blue circles) plotted with theoretical reduced scattering values as predicted using Mie Theory (orange line). (**b**) Measured absorption coefficient of methylene blue at a concentration of 10.8 mmol/L in deionized water at 293 K (blue circles) plotted with theoretical values from the model proposed by Fernandez-Perez et al.[47]
